# IMPAACT 2041: Designing the Study of Glecaprevir/Pibrentasvir Treatment During Pregnancy

**DOI:** 10.1093/ofid/ofag537

**Published:** 2026-08-28

**Authors:** Ravi Jhaveri, Kristina M Brooks, Vincent Mainella, John Marcinak, Mong-Jen Chen, Katie McCarthy, Camlin Tierney, Zhongli J Zhang, Bryan S Nelson, Patricia M Flynn, Nathan Furukawa, Ahizechukwu C Eke, IMPAACT 2041 Protocol Team, Renee Browning, Haley Brozik, Margaret Burroughs, Christine Chiou, Maria Corcorran, Emma Duffy, Molly Dyer, Alexandru Iacob, Cheryl Jennings, Vindya Kakarla, Jatinder Kaur, Sonia Lee, Philip Marzinek, Andrea McMunn, Talia Pindyck, Chitara Rowry, Hannah Small, Leah Spalding, Chelsea Stotz, Franklin Yates

**Affiliations:** Division of Infectious Diseases, Ann & Robert H. Lurie Children’s Hospital of Chicago, Illinois, USA; Department of Pediatrics, Northwestern University Feinberg School of Medicine, Chicago, Illinois, USA; Department of Pharmaceutical Sciences, Skaggs School of Pharmacy and Pharmaceutical Sciences, University of Colorado Anschutz Medical Campus, Aurora, Colorado, USA; Department of Pharmaceutical Sciences, Skaggs School of Pharmacy and Pharmaceutical Sciences, University of Colorado Anschutz Medical Campus, Aurora, Colorado, USA; Global Patient Safety, AbbVie Inc., North Chicago, Illinois, USA; Clinical Pharmacology, AbbVie Inc., North Chicago, Illinois, USA; FHI 360, Durham, North Carolina, USA; Center for Biostatistics in AIDS Research, Harvard T. H. Chan School of Public Health, Boston, Massachusetts, USA; Center for Biostatistics in AIDS Research, Harvard T. H. Chan School of Public Health, Boston, Massachusetts, USA; Center for Biostatistics in AIDS Research, Harvard T. H. Chan School of Public Health, Boston, Massachusetts, USA; Department of Infectious Diseases, St. Jude Children's Research Hospital, Memphis, Tennessee, USA; National Center for HIV/AIDS, Viral Hepatitis, STD, and TB Prevention, Centers for Disease Control and Prevention, Atlanta, Georgia, USA; Division of Maternal Fetal Medicine, Department of Gynecology and Obstetrics, Johns Hopkins University School of Medicine, Baltimore, Maryland, USA

**Keywords:** glecaprevir, Hepatitis C, pibrentasvir, pregnancy, treatment

## Abstract

The significant increase in hepatitis C virus (HCV) infection prevalence among young adults, including those who may become pregnant, has led to a corresponding increase in the number of infants exposed to and infected with HCV. Despite a recommendation for universal HCV screening during pregnancy, there is not always a clear pathway to immediately initiate treatment for maternal cure, which could also prevent vertical transmission like the approach for human immunodeficiency virus (HIV) or hepatitis B virus. In 2019, the American Association for the Study of Liver Diseases/Infectious Diseases Society of America HCV Guidelines panel endorsed the use of shared decision making between providers and patients when considering HCV treatment during pregnancy. Direct-acting antivirals, including glecaprevir/pibrentasvir (GLE/PIB), have been tremendously effective in curing >95% of individuals who begin treatment along with an overall well-characterized safety profile, but data supporting their use when initiated during pregnancy are more limited. International Maternal Pediatric Adolescent AIDS Clinical Trials 2041 is a multicenter single-arm phase I/II clinical trial designed to evaluate the pharmacokinetics and safety of GLE/PIB treatment initiated in pregnant women with HCV mono-infection or HCV/HIV co-infection. In this paper, we describe the relevant background data assembled from various sources to write the protocol and design the study.

## Teaser

There has been an unmet need for research into treatment of hepatitis C during pregnancy. This paper describes planning the first pharmacokinetic clinical trial using glecaprevir/pibrentasvir in pregnant women.

The landscape of hepatitis C virus (HCV) infections in the United States has changed dramatically over the last 15 years [1, 2]. As a result of widespread injection drug use, what was once an infection primarily of older adults has become increasingly prevalent in adolescents and young adults? From 2006 to 2012, acute HCV infection case rates in young adults increased in some regions of the country by 200%–400% [3]. The significant increase in HCV prevalence among young adults, including those who may become pregnant, has led to a corresponding increase in the number of infants exposed to and infected with HCV. Approximately 3%–8% of perinatally exposed infants acquire HCV infection, of whom 3%–5% of children progress to chronic infection [4–8]. Human immunodeficiency virus (HIV) co-infection is an important risk factor that can significantly increase HCV transmission if untreated [9]. Testing recommendations in young adults and, specifically in those who are pregnant, have evolved over several years, with the American Association for the Study of Liver Diseases (AASLD)/Infectious Diseases Society of America (IDSA) HCV Guidelines panel (2018), the Centers for Disease Control and Prevention (CDC) (2020), Society for Maternal-Fetal Medicine (SMFM) (2021), and the American College of Obstetricians and Gynecologists (ACOG) (2021) all now recommend HCV screening during each pregnancy [10–14].

Despite advances in HCV screening during pregnancy, there is not always a clear pathway to immediately initiate treatment while patients are actively engaged in care and are often at high-risk of falling out of care after delivery [15]. Direct-acting antivirals (DAAs), including glecaprevir/pibrentasvir (GLE/PIB), have been effective in curing >95% of individuals who begin treatment along with an overall well characterized safety profile. However, none of the regimens were initially studied during pregnancy [16], though recently there is increasing pharmacokinetic (PK) and efficacy data with sofosbuvir/velpatasvir (SOF/VEL) use in pregnancy [17–19]. Initiating treatment during pregnancy could offer many benefits: patients are actively engaged in care; health insurance is more comprehensive and extends to 1 year postpartum; ability to complete therapy in the third trimester with sustained virologic response (SVR) testing at delivery; elimination of maternal viremia reduces risk of vertical transmission to the infant(s), and avoids deferring treatment to the postpartum period where conflicting priorities result in poor treatment initiation rates [20, 21].

There have been efforts to make initiating HCV treatment during pregnancy more viable option [22]. In 2019, the AASLD/IDSA HCV Guidelines panel endorsed the use of shared decision making between providers and patients when considering HCV treatment during pregnancy [23]. In 2019 and 2020, phase 1 studies of SOF/VEL were initiated to evaluate the PK of these drugs during pregnancy. In comparison to nonpregnant adults, SOF exposures were higher and the nucleoside analog (GS-331007) exposures were decreased, while VEL exposures were comparable or slightly lower, and thus no dose adjustments were required [18, 24]. In 2022, a follow-up phase 4 multicenter study of SOF/VEL (STORC) was initiated to evaluate the efficacy of SOF/VEL hepatitis C treatment during pregnancy, with 83 participants through March 2026, demonstrating 63 (96%) maternal sustained virologic response 12 weeks postdosing (SVR12) and no cases of perinatal transmission [18]. The 2 treatment failures to date missed a significant number of doses while also having re-exposure to HCV. While SOF/VEL is an important option for HCV treatment, data are only now emerging using the other major pan-genotypic DAA option, GLE/PIB [25]. The differing pharmacology and drug class of this regimen combined with its shorter treatment duration necessitates further research during pregnancy.

The International Maternal Pediatric Adolescent AIDS Clinical Trials (IMPAACT) 2041 (NCT07040319) is a multicenter single-arm phase I/II clinical trial designed to evaluate the PK and safety of GLE/PIB treatment initiated in pregnant women with HCV mono-infection or HCV/HIV co-infection. In this paper, we describe the relevant background assembled from various sources to write the protocol and design the study.

## GLE/PIB REVIEW OF EFFICACY, SAFETY, AND PK IN NONPREGNANT PATIENTS

GLE is a nonstructural (NS) protein 3/NS protein 4A (NS3/NS4A) protease inhibitor of HCV, while PIB is an NS protein 5a (NS5A) inhibitor. Marketed under the brand name MAVYRET, GLE/PIB has pangenotypic activity and is a fixed-dose combination of 100 mg/40 mg film-coated bilayer tablets administered as 3 tablets taken at the same time once daily with food [26]. The efficacy and safety of GLE/PIB have been well established in nonpregnant populations such as otherwise healthy children, adolescents and adults; adults with compensated cirrhosis; severe renal impairment or end-stage renal disease; and patients with HIV co-infection and adults with acute HCV infection [27–32]. The key benefits of treatment with the GLE/PIB regimen are high sustained virologic response 12 weeks postdosing (SVR_12_) rates (>97%) across all genotypes and subpopulations along with a shorter duration of treatment (8-week duration) than that of SOF/VEL (12 weeks). Data from large clinical trials and real-world studies demonstrate that GLE/PIB is well tolerated across different populations, with fewer than 0.1% of patients experiencing a serious adverse event related to GLE/PIB, and no significant hepatotoxicity observed [33].

The time to peak drug concentrations (T_max_) of GLE and PIB occur 3–5 h and 5 h after dosing. Mean GLE and PIB exposures increase by 163% and 40% with moderate fat meals, and by 83% and 53%, respectively, with high fat meals. Based on available data, GLE/PIB should be taken with food, without regard to calorie or fat content. Approximately 97.5% and 99.9% of GLE and PIB, respectively, bind to human plasma proteins. GLE exhibits limited metabolism in vitro, predominantly by CYP3A4/5 and to a much lesser extent by CYP2D6, CYP2C9, and CYP2C8. Metabolism plays no role in the elimination of PIB. Both GLE and PIB are predominantly excreted through the biliary-fecal route with 92.1% and 96.6% of the administered dose recovered in the feces, respectively. Less than 1% of either drug is excreted in the urine. The terminal elimination half-lives of GLE and PIB are approximately 6 and 13 h, respectively [34].

Exposure–virologic response analyses evaluated demographic variables (age, sex, weight, and race), baseline HCV RNA viral load, IL28, GLE, and PIB area under the concentration–time curve from 0 to 24 h at steady-state (AUC_24,ss_), renal impairment status, co-infection with HIV, coadministration with RBV, and treatment duration were tested, and found higher PIB AUC_24,ss_ was associated with an increased likelihood of achieving SVR12 [34].

## PHYSIOLOGICAL AND SAFETY CONSIDERATIONS OF GLE/PIB PK AND TREATMENT DURING PREGNANCY

Physiological changes during pregnancy could potentially alter GLE/PIB absorption, distribution, metabolism, and elimination [34, 35]. Delayed gastric emptying, increased gastric pH, and prolonged gastrointestinal transit time may impact GLE/PIB absorption. GLE/PIB is also better absorbed with meals in comparison to the fasted state, but eating patterns may vary during pregnancy. Both GLE and PIB are substrates and inhibitors of the efflux transporter, P-glycoprotein, and P-glycoprotein expression may also be altered in pregnancy. These collective factors may result in differing C_max_, T_max_, or total drug exposures (ie area under the concentration-time curve [AUC]). Increased total body water and plasma volume may increase GLE/PIB apparent volume of distribution. Decreased albumin concentrations may impact GLE/PIB total plasma and unbound fractions, and GLE/PIB plasma free drug concentrations could be decreased if intrinsic clearance is increased. Increased liver blood flow and altered activities of drug-metabolizing enzymes, especially increased CYP3A4 activity, may impact GLE/PIB hepatic clearance. These collective changes are also dynamic throughout pregnancy, and thus steady-state exposures achieved in nonpregnant adults may not be the same during pregnancy, making the PK of GLE and PIB critically important to characterize.

### Reproductive Toxicology

Both embryofetal development (EFD) and perinatal/postnatal development (PPND) studies are required in the evaluation of the risks of adverse effects in pregnancy for drugs in clinical development [36]. Based on results of the EFD and PPND studies following oral administration with either GLE or PIB to laboratory animals, there are no anticipated risks for reproductive toxicity. High multiples of clinical exposures were not associated with any adverse effects in the rodent (rat for GLE and mouse for PIB). There were no malformations or variations in rabbits with GLE, though maternal toxicity (decreased food consumption and body weight loss) precluded being able to achieve clinically efficacious exposures. There were no embryo-fetal effects at the maximal feasible exposure to GLE in the rat [32]. With PIB, there were no embryofetal effects at the maximal feasible exposure in the rabbit [32].

## RATIONALE AND ADVANTAGES OF USING THE IMPAACT NETWORK

The IMPAACT Network (https://www.impaactnetwork.org/) has been instrumental in advancing research on HIV treatment and perinatal prevention, particularly among pregnant women. Established in 2006 through the merger of the Pediatric AIDS Clinical Trials Group and the perinatal scientific working group of the HIV Prevention Trials Network, IMPAACT has created a robust infrastructure for conducting comprehensive studies during pregnancy and postpartum periods. With over 40 clinical research sites worldwide, including in high HIV prevalence regions, IMPAACT ensures that its studies are representative and applicable across various settings. Leadership at each site is a team effort between Pediatric Infectious Diseases, Adult Infectious Diseases, and Maternal-Fetal Medicine/Obstetrics. The Network’s commitment to understanding the PK, safety, and efficacy of treatments initiated or continued during pregnancy has led to significant insights that inform clinical guidelines and practices [37–55]. Furthermore, IMPAACT's commitment to community engagement and ethical research practices ensures that the voices and needs of pregnant women with HIV are central to its research endeavors. A brief summary of key studies and insights from IMPAACT is shown in Table 1.

**Table 1.** Summary of Key IMPAACT Studies Relevant to Pregnancy

Study	Phase	Sample Size	Focus	Key Contributions
IMPAACT P1026 s/2026 [39, 41–44]	IV	1784 pairs	Pharmacokinetics of antiretroviral drugs in pregnancy	Informed dosing and safety profiles of ARVs during pregnancy; IMPAACT 2026 supported FDA label update for BIC/FTC/TAF use in the second and third trimesters and postpartum
IMPAACT 1077BF/1077FF (PROMISE) [45–48]	III	3529 pairs	Prevention of perinatal and breastfeeding HIV transmission	Evaluated optimal ART strategies to reduce mother-to-child HIV transmission and support maternal health across pregnancy and postpartum
IMPAACT 2010 (VESTED) [49–51]	III	643 pairs	Comparative safety and efficacy of DTG- vs EFV-based regimens	Demonstrated superior outcomes of dolutegravir-based regimens in pregnant and postpartum women and their infants
IMPAACT 2032 [40]	IV	53 women	Remdesivir pharmacokinetics and safety during COVID-19	Provided pivotal data leading to FDA approval of remdesivir for use in pregnancy during the COVID-19 pandemic
IMPAACT 2009 [37, 38]	NA	390 pairs	Pre-exposure prophylaxis for HIV in pregnancy	Evaluated PK, acceptability, and safety of PrEP in pregnant adolescents and women with HIV
IMPAACT P1025 [52, 53]	NA	3090 pairs	Prospective observational cohort of pregnant women with HIV	Characterized maternal and infant outcomes associated with ART use during pregnancy, guiding clinical care, and long-term safety assessments
IMPAACT P1078 (TB APPRISE) [54, 55]	IV	956 pairs	Timing and safety of isoniazid preventive therapy (IPT)	Influenced WHO guidelines by demonstrating increased adverse events when IPT was initiated during pregnancy vs postpartum.

Abbreviations: ART, antiretroviral therapy; ARVs, antiretroviral drugs; BIC/FTC/TAF, bictegravir/emtricitabine/tenofovir alafenamide; DTG, dolutegravir; EFV, efavirenz; FDA, U.S. Food and Drug Administration; HIV, human immunodeficiency virus; IPT, isoniazid preventive therapy; PK, pharmacokinetics; PrEP, pre-exposure prophylaxis.

The IMPAACT Network’s prior track record of clinical research leading to FDA and EMA label changes makes it uniquely qualified to conduct IMPAACT 2041 focusing on HCV treatment during pregnancy. Similar to the experience with the IMPAACT PK studies of EVG/c/FTC/TAF and remdesivir in pregnancy, data from IMPAACT 2041 may support an updated FDA label describing the safety and PK of GLE/PIB in pregnancy.

## CONSIDERATIONS IN TRIAL DESIGN

**Figure 1.**
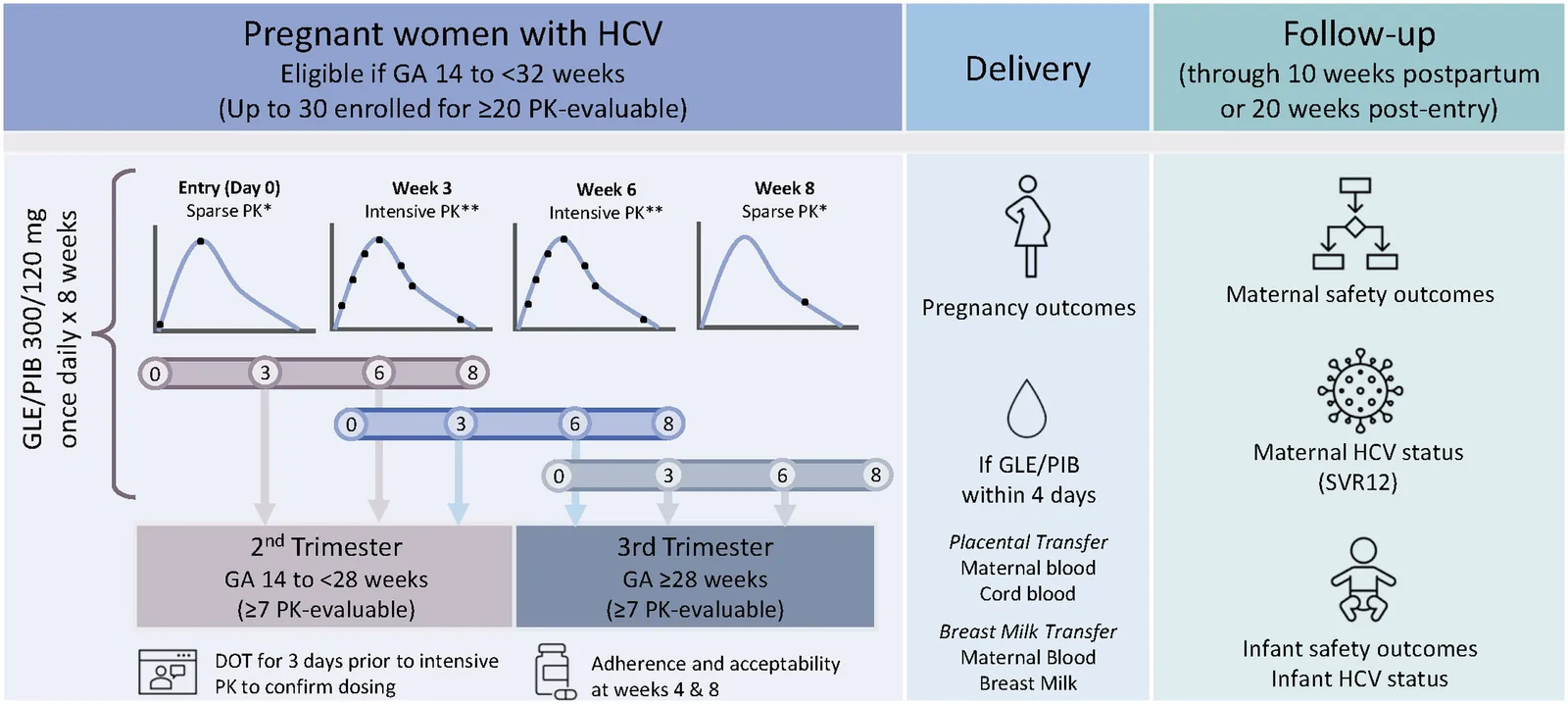
Overview of the IMPAACT 2041 study design. Sparse PK sampling on day 0 at predose (time 0) and 2–4 h postdose and at week 8 is convenience sampling (if GLE/PIB dose within 4 d). Intensive PK sampling will take place at time 0 (predose), 1, 2, 3, 4, 5, 6, 8, 10, and 24 h postdose. Participants may elect to complete only one intensive PK and one abbreviated PK, with samples collected at time 0 (predose), 2, and 4 h postdose. Abbreviations: DOT, directly observed therapy; HCV, hepatitis C virus; GA, gestational age; GLE, glecaprevir; PIB, pibrentasvir; PK, pharmacokinetic; SVR12, sustained virologic response at 12 weeks post-treatment.

An overview of the study design is detailed in Figure 1. An open-label design without an untreated concurrent control group is the accepted standard by the FDA for conducting a study in this population [56]. In this study, the comparison group for PK analyses will be historical, nonpregnant women between 18 and 45 years of age and treated with GLE/PIB in prior phase 2/3 studies. The study will be conducted among up to 30 pregnant participants and their infants to achieve at least 20 PK-evaluable pregnant participants. The sample size of 20 PK-evaluable participants was not selected to achieve a desired level of statistical precision for safety, but rather to acknowledge the known challenges of enrolling large numbers of patients into any study during pregnancy and to satisfy the pharmacological objective of the study [18, 40, 57]. Further rationale for the PK targets is presented in a later section, and the total sample size was selected to account for anticipated nonevaluability and losses to follow-up. In addition to the PK assessments during pregnancy and postpartum period, optional exploratory sample collections at delivery to assess placental and breast milk transfer may also be performed if the mother is still on GLE/PIB.

Pregnant participants will be screened for eligibility and enrolled between 14 and 32 weeks of gestation. The rationale for this specific treatment initiation window is to allow for the completion of fetal organogenesis that occurs throughout the first trimester. It also represents the gestational age that most women diagnosed with HCV during prenatal screening would present for treatment. The upper limit of 32 weeks was chosen to ensure that participants have adequate time to complete the 8-week course of GLE/PIB therapy by full term (40 weeks), thereby maximizing the likelihood of achieving virologic cure prior to delivery. Upon enrollment, pregnant participants will take GLE/PIB once daily for 8 weeks. Postpartum women may be followed through 10 weeks postpartum or 20 weeks postentry, whichever is longer, depending on gestational age at entry and timing of delivery. Infants will be followed at a minimum of 12 weeks following GLE/PIB exposure. If participants are taking GLE/PIB postpartum and during lactation, breast milk samples will be collected. Overall, depending on gestational age at entry, participants over the life of the study will complete 6–8 study visits.

### Study Objectives

The primary objectives of the study are: (1) to describe the PK of GLE and PIB initiated in pregnant women with hepatitis C, compared to historical data from nonpregnant female adults with hepatitis C and (2) to describe the safety of GLE/PIB initiated in pregnant women with hepatitis C while on treatment and through 10 weeks postpartum (or 20 weeks post-treatment initiation) [58]. The clinical questions to be addressed with the primary objectives are: (1) what is the geometric mean ratio comparing plasma AUC_0-24h_ of GLE and PIB in pregnant women with HCV infection on daily GLE/PIB vs nonpregnant female adults? and (2) what is the probability that a woman who initiates treatment for HCV during pregnancy on/after 14 weeks gestation experiences at least 1 adverse event related to co-formulated GLE/PIB while taking GLE/PIB and through the latter of 10 weeks postpartum or 20 weeks post-treatment initiation? Secondary objectives include evaluating the SVR_12_ among pregnant women, measuring the frequency of perinatal HCV transmission, and describing pregnancy outcomes and safety of GLE/PIB in infants through 10 weeks of life.

### Timing of HCV Testing, Screening, Enrollment and Completion of 8-Week Course of Therapy

The pregnant/postpartum participants will have HCV ribonucleic acid (RNA) testing first at the screening visit and again at visit weeks 3, 8, and 20; additional HCV RNA testing may also be completed at the delivery and week 10 postpartum visits, depending on prior collection and timing of these visits in relation to time on GLE/PIB. Infants will complete study visits at birth and at week 10, with HCV RNA testing performed at week 10 and, as needed, 12 weeks after last GLE/PIB exposure *in utero* or through breast milk.

## STUDY OUTCOMES AND PK ASSESSMENTS

### Selection of PK Targets

**Figure 2.**
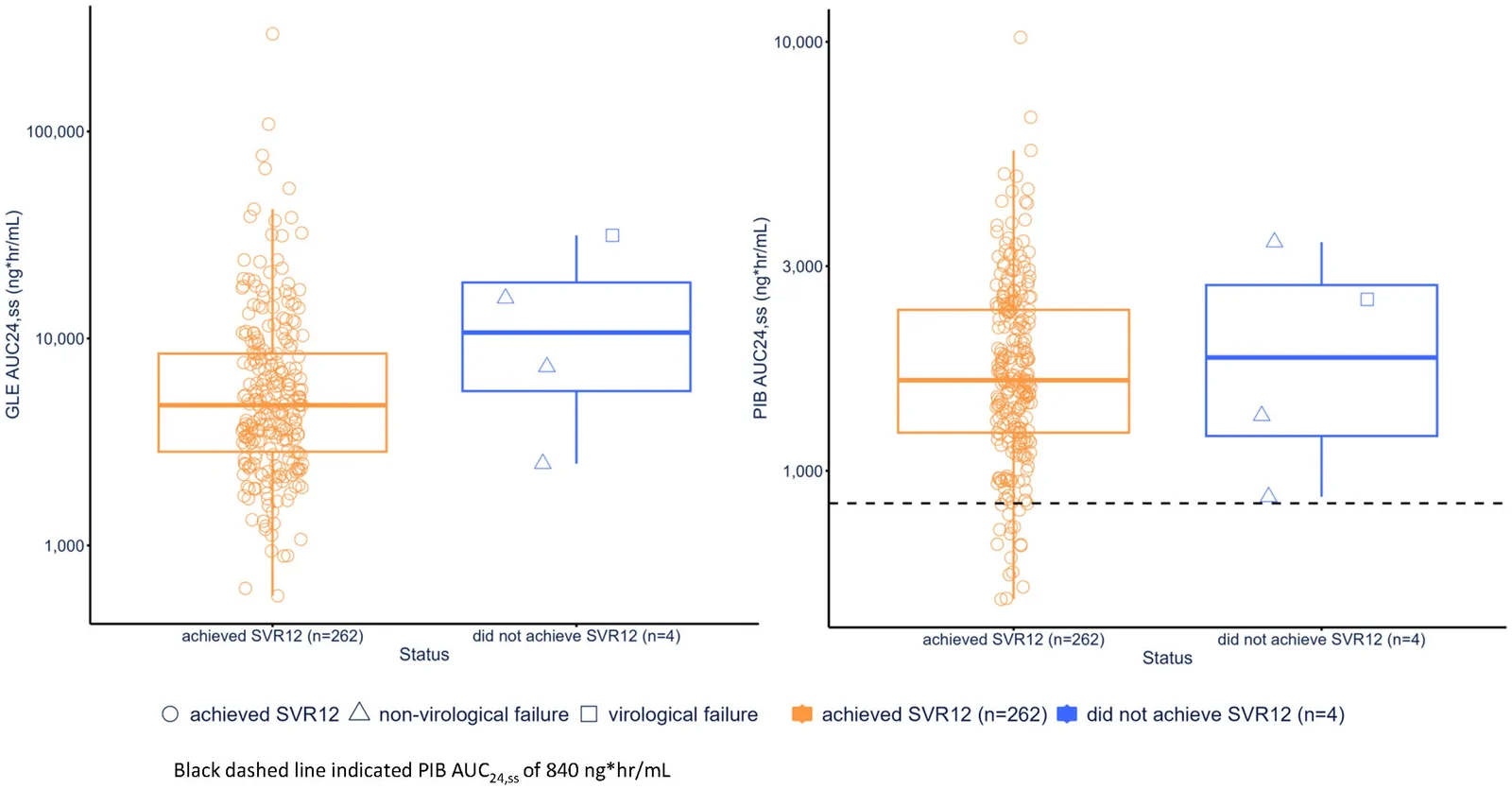
Distributions of model-predicted GLE and PIB AUC_24,ss_ for nonpregnant women age ≥18–≤45 with chronic HCV infection from previous phase 2/3 studies. PK values for GLE (left) and PIB (right) are shown here. The PK target for this study, PIB 840 ng h/mL is shown with the dashed line. Abbreviations: AUC_24,ss_, area under the concentration-time curve from 0 to 24 h at steady state; GLE, glecaprevir; HCV, hepatitis C virus; PIB, pibrentasvir; PK, pharmacokinetics.

The exposure-response model (ie exposure–SVR12 model) for GLE/PIB identified PIB AUC as the only significant predictor for SVR12 in participants treated with this combination. In the model, GLE exposure was tested, but not significant, and GLE and PIB exposures were highly correlated. Efforts to find lower efficacy bounds for GLE were inconclusive as no clear trend was observed [34]. To help inform PK target selection, model-predicted GLE/PIB AUC_0-24h_ values in nonpregnant women were simulated using previously developed population PK models from phase 2/3 studies [59]. Data were available in 266 nonpregnant women aged 18–45 years to provide an initial starting point of drug exposures associated with treatment efficacy, and GLE and PIB AUC_0-24h_ values were simulated for each participant to support the generation of a historical control population for comparison with during pregnancy. A total of 262 out of 266 women achieved SVR_12_ in this population. For the remaining 4 women, 3 did not achieve SVR_12_ due to reasons other than virologic failure, and 1 woman categorized as virologic failure had prior DAA treatment experience and only completed 46 days treatment (Figure 2). Furthermore, exclusion of these women did not significantly impact central estimates of AUC_24,ss_ in this population. From the simulation results, the geometric mean PIB AUC_24,ss_ in nonpregnant women aged 18–45 years old (*n* = 262) was 1680 ng h/mL.

Decreases in PIB AUC_24,ss_ by 50% have been associated with a less than 1% decrease in SVR_12_ in nonpregnant treatment-naïve and -experienced persons with genotypes 1, 2, 4, 5, or 6 HCV infection, ultimately representing limited clinical significance [34]. The same magnitude of decrease in PIB AUC_24,ss_ is associated with 3.3% decrease in SVR_12_ rate in those with genotype 3 infection, and the SVR_12_ rate drops below 90% in this population when PIB AUC_24,ss_ decreases by 70%. Additionally, exposures within 0.5- to 2-fold of population geometric mean steady-state AUC_24,ss_ values were previously used as the target efficacious exposure range in the GLE/PIB DORA Part 2 study of children 3–<12 years old [29]. Thus PIB AUC_24,ss_ of 840 ng h/mL, reflecting a 50% decrease from the prior geometric mean, was selected as a reference threshold for comparison in IMPAACT 2041 pregnant/postpartum participants.

### Sample Size Justification

Using the geometric mean ratio thresholds above, precision estimates were calculated to assess the ability to detect differences in the AUC_24,ss_ of either GLE or PIB in pregnant vs nonpregnant women ranging from a 70% decrease to 200% increase. These calculations were performed across sample sizes of 7 (for subgroup analyses by trimester or HIV status), 14 (intermediate size), and 20 participants (the overall target size) using 2-sided 90% confidence intervals that were then calculated across a range of log-transformed standard deviations from historical controls ranging from 0.5 (approximate to PIB) to 1.5 (approximate to GLE). A sample size of 20 was adequate to exclude no difference for both drugs if the estimated ratios fell outside 50%–200%. For subgroup analyses, a sample size of 7 was adequate to exclude no difference if the estimated ratios fell outside 50%–200% for a natural log standard deviation of 0.9 or less.

### PK Sampling Design and Analysis

Traditional pregnancy PK studies of chronically administered medications often use a longitudinal design, in which participants are sampled during pregnancy and again in the postpartum period to allow for within-participant comparisons. However, because the treatment duration for GLE/PIB is only 8 weeks, this approach was not feasible. The brief course of therapy precludes postpartum PK assessments and limits between-trimester sampling, necessitating alternative PK study designs with a visit schedule relative to treatment initiation as opposed to a gestational age/postdelivery linked schedule. As the inclusion criteria span pregnant women 14–32 weeks gestation, this permits the ability to enroll participants across different stages of the second and third trimester to better understand drug exposure and capture PK variability across trimesters and will help inform population PK model development in pregnant women.

PK assessments comprise sparse and intensive PK sampling at select times throughout the 8-week treatment period. On the first day of study drug dosing, samples will be collected predose and 2–4 h postdose. Intensive PK evaluations consisting of 10 measurements over a 24-h period will be conducted at weeks 3 and 6 (ie steady-state) to characterize full concentration–time curves across a variety of gestational ages. To facilitate participant accrual and circumvent potential logistical challenges, an abbreviated sparse PK visit (predose, 2-h and 4 h postdose) is permitted in place of one of the intensive PK assessments at weeks 3 or 6. A single convenience PK sample will then be collected at the Week 8 visit to then permit the ability to potentially assess washout PK. Samples for plasma protein binding assessments will also be collected at each set of PK visits. Directly observed therapy through in-person or video-based methods are also required for the 3 days leading up to the intensive PK assessment to ensure that nonadherence does not impact these assessments. An interim review will be performed once 5 participants are enrolled with at least 1 intensive PK assessment completed and assessed as PK evaluable to perform an initial check of GLE/PIB exposures. The main parameter of interest during this initial check is PIB AUC_0-24h_, with values falling below 840 ng h/mL prompting study re-evaluation. However, all available PK data for both GLE and PIB, safety data from pregnant/postpartum participants and their infants, and SVR_12_ data (if available) will be included in the interim review.

In addition to the intensive PK sample collection during pregnancy, samples will also be collected to assess placental and breast milk transfer of GLE/PIB. These may occur at the delivery visit if the mother has taken a dose of GLE/PIB within the previous 4 days. For placental transfer, paired cord blood and maternal peripheral blood samples will be collected to determine cord blood-to-maternal ratios. For breast milk transfer, a paired breast milk and maternal peripheral blood sample will be collected to calculate breast milk-to-maternal blood ratios and generate estimates of relative infant doses based on breast milk concentrations. Postpartum PK assessments may also be performed in alignment with the planned PK visits depending on when a participant delivers during the GLE/PIB treatment course.

### SVR_12_ Analysis of Pregnant Participants

The analysis of SVR_12_ will include all pregnant/postpartum participants who received at least 1 dose of GLE/PIB (initiated treatment set). If a pregnant/postpartum participant does not have any HCV RNA measurements in this prescribed time window and unless there are preceding and subsequent HCV RNA measurements that are both undetected, they will be considered as not achieving SVR_12_. An additional supplementary analysis will include only pregnant/postpartum participants who completed the intended 8-week GLE/PIB treatment and had an HCV RNA sample collected at week 20 (completed treatment SVR_12_ set).

### Safety Outcomes of Interest

As written in the protocol, the study will evaluate safety (1) while on study drug and (2) after initiating treatment through the latter of 10 weeks postpartum or 20 weeks post-treatment initiation. Outcomes of interest include grade 3 or higher adverse events (AEs) (DAIDS classification) assessed as related to study drug and serious AEs assessed as related to study drug. Pregnancy outcomes are part of the secondary outcomes of interest and include complications of pregnancy identified following enrollment and before delivery; date and time of onset of labor; type of labor (spontaneous or induced); mode of delivery; complications of delivery; outcome of delivery.

### Infant HCV RNA Testing

The CDC recommendation for testing infants exposed to HCV was recently revised to HCV RNA testing at 2–6 months of life [4, 60]. For this study, infant follow-up testing was reconciled with the timing of the postpartum visits. These usually occur around 6 weeks after delivery, which is before the recommended window. To allow for infant testing to fall within the CDC recommended window, the postpartum study visit will be at 10 weeks. This window between 10–14 weeks postpartum allows for appropriate capture of HCV RNA data for infants for HCV/HIV exposed infants' appropriate HIV testing and CD4 T-cell measurement if applicable and offers significant flexibility for the sites and participants to schedule the in-person visit at a convenient time. In the rare event that GLE/PIB treatment is completed near delivery, infants will have an additional follow-up visit with HCV RNA testing to fulfill the 12-week postexposure assessment.

## CONCLUSIONS

With the recent increased interest in expanding HCV elimination efforts, IMPAACT 2041 will provide key data with GLE/PIB treatment during pregnancy. Combined with the results from the SOF/VEL in pregnancy study underway, these data will be crucial for the development of professional society guidelines from AASLD/IDSA, ACOG and SMFM. A recommendation to treat HCV infection during pregnancy could unlock a more effective model of care—one that not only advances global HCV elimination efforts, but also significantly reduces, and potentially eliminates vertical transmission both in the United States and abroad.
